# Comparative Analysis of Crystal Violet-Binding Aptamers as Potential Cores for Binary Sensors

**DOI:** 10.3390/ijms26199833

**Published:** 2025-10-09

**Authors:** Gleb A. Bobkov, Gleb S. Yushkov, Andrei D. Kuzmin, Tatiana D. Popysheva, Elena I. Stepchenkova, Maria S. Rubel

**Affiliations:** 1 Laboratory of Amyloid Biology, Saint-Petersburg State University, 199034 Saint Petersburg, Russia; gleb.bobkov@spbu.ru (G.A.B.); andrei.kuzmin.itmo@gmail.com (A.D.K.); 2 Center for Molecular and Biological Technologies, ITMO University, 191002 Saint Petersburg, Russia; yushkov@scamt-itmo.ru (G.S.Y.); tanya.popyscheva@yandex.ru (T.D.P.); 3 Vavilov Institute of General Genetics, Saint Petersburg Branch, Russian Academy of Sciences, 199034 Saint Petersburg, Russia

**Keywords:** crystal violet, aptamer, ‘light-up’ aptamer, CV30S, G-quadruplex, binary sensor

## Abstract

‘Light-up’ aptamers are short oligonucleotides that can induce fluorescence of certain organic compounds upon binding. In this study, we compared three crystal violet (CV) aptamers—CV30S, parallel G-quadruplex (G4), and antiparallel G4—regarding their absolute fluorescence intensity, signal-to-background ratio (S/B), and potential as a core component in binary sensors for nucleic acid detection. The G4 antiparallel aptamer exhibited the highest fluorescence intensity and a robust S/B ratio, indicating its effectiveness in stabilizing the CV binding and enhancing fluorescence. In contrast, the G4 parallel aptamer demonstrated poorer performance, suggesting that its structural topology is less suitable for interactions with CV. The CV30S aptamer showed distinct advantages in binary sensor configurations, achieving the best limit of detection at 6 nM.

## 1. Introduction

**Figure 1:**
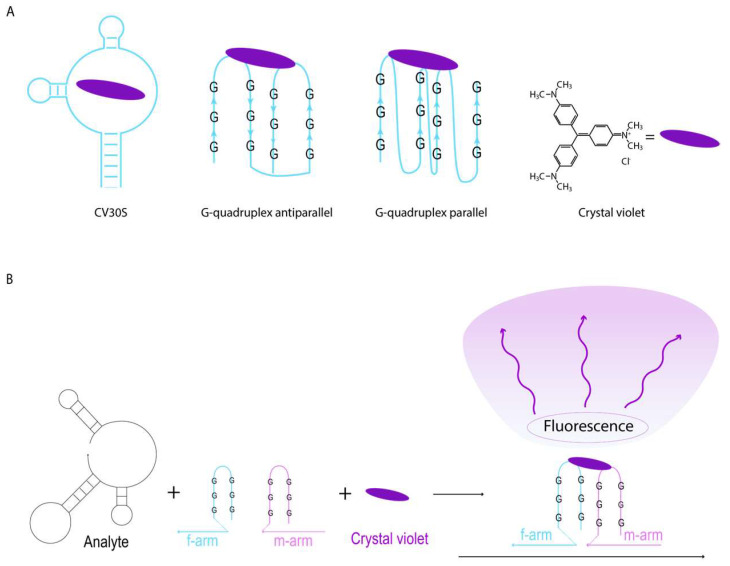
The principal scheme of the CV-binding aptameric binary sensors with different light-generating aptamers. Three CV-binding aptamers used in the study (**A**). An example of the binary light-emitting complex based on the antiparallel G-quadruplex aptameric core (**B**).

Aptamers are short, single-stranded oligonucleotides selected in vitro for their ability to bind specific targets with high affinity and specificity [1,2,3]. Their stability, ease of synthesis, and tunable secondary structures make them attractive components in biosensing technologies as alternatives to antibodies [4,5,6]. In addition to compound-recognition aptamers, there is a specific class of aptamers called ‘light-up’ aptamers (Figure 1A). These oligonucleotides can cage organic compounds and redistribute electron densities within them, resulting in fluorogenic properties [7]. Such ‘light-up’ aptamers are novel tools with great potential for probe design and in-cell visualization [8,9]; therefore, it is relevant to search for the most effective combinations of aptamer and fluorescent dye.

‘Light-up’ aptamers can be integrated into signal visualization systems by coupling them with sensing elements (analyte-detecting arms) [10]. The ‘light-up’ aptamer can function either as an entire molecule or split into two parts, creating a binary sensor configuration [11] (see Figure 1B). In binary sensors, the analyte-detecting arms bind the analyte, bringing the two separated halves of the core structure in closer proximity. Only close proximity of binary sensor parts allows the core to capture the dye molecule from the solution and create a detectable fluorescence signal. This approach is exceptionally effective for nucleic acid sensing due to its high selectivity, capability to deal with folded nucleic acids, and ease of DNA construct design [12].

‘Light-up’ aptamers are often designed for use with rare and expensive dyes such as dapoxyl [13] or 3,5-difluoro-4-hydroxybenzylidene imidazolinone [14]. Crystal violet (CV), a triarylmethane dye, in its turn, has been widely used in biotechnology for cell viability assays, bacterial staining, and quantification of biofilms due to its strong interaction with biological macromolecules [15,16]. This dye possesses a number of properties that make it an excellent candidate as a dye component in ‘light-up’ aptamers. CV is characterized by inherently low fluorescence in aqueous solutions; however, its fluorescence can be significantly enhanced when CV binds to certain aptamers, such as CV30S or G-quadruplexes (G4) [17,18,19]. An additional advantage of using this dye as a reporter molecule in fluorescence-based assays is its low cost and wide availability, making it highly suitable for diagnostic applications.

Three ‘light-up’ aptamers were found to be bound to CV. First, CV30S is a recently developed ‘light-up’ aptamer that exclusively binds CV and emits light at 640 nm (580 nm ex), with K_D_ = 0.49 ± 0.04 µM [19]. However, CV30S has not yet been tested as part of an effective sensor, and only non-selective G4 structures have been used with CV to achieve fluorescence [20].

Another type of CV-binding sequences includes guanine-rich sequences capable of forming G4 structures. They efficiently bind to CV with K_D_ −1.06 µM [21,22]. Planar organic compounds, including CV, bind to the lateral sides of the G4, leading to redistribution of electronic densities [23], that can be beneficial for their enzymatic properties (e.g., hemin) or fluorescence (e.g., CV or thioflavin T) [24,25,26]. G4 aptamers can adopt different topologies—predominantly parallel and antiparallel—depending on their sequence and environmental factors such as ionic strength and pH [27,28,29]. These structural variations affect both the affinity for CV and the magnitude of the resulting fluorescence enhancement. G4 is not selective for planar organic compounds and is widely used in biosensing applications [30]. CV–G4 systems can be employed for the detection of metal ions and the characterization of ionic compositions in urine samples, offering a label-free and low-cost alternative to conventional detection methods [31].

The aim of this study is to perform a systematic comparison of three CV-binding ‘light-up’ aptamers—parallel G4, antiparallel G4, and CV30S—as cores for binary sensors. The study involves analyzing the performance of two aptamer–dye systems: one that utilizes a single molecule aptamer and another that employs a split aptamer as part of binary sensors at different temperatures. We have assessed the suitability of different aptamer structures for CV-based fluorescence sensing and demonstrated their potential in nucleic acid detection applications by incorporating them into binary sensors for a model sequence of *Aeromonas hydrophila*. This bacterial species is a conditional fish pathogen that can be harmful when it proliferates uncontrollably. The choice of the analyte was determined by the need for effective monitoring of disease outbreaks and methodologies utilizing aptamers are well aligned with these requirements. During an epidemic event, obtaining samples rich in pathogen DNA becomes relatively straightforward, eliminating the requirement for preliminary amplification steps. Consequently, direct identification of the causative agent via aptamer-based methods proves both feasible and advantageous.

## 2. Results

### 2.1. Buffer Optimization

To determine the optimal buffer composition that maximizes the efficacy of each of the three aptamer-CV complexes (thereby unlocking their full potential for sensor applications), we assessed fluorescence intensity and the signal-to-background ratio in buffers with varying pH (from 6 to 7.5) and potassium concentrations (from 0 to 112.5). The concentration of potassium ions and pH of the reaction mixture critically influence the formation and stabilization of G-quadruplex confirmation, unlike sodium or other salts [32,33,34]. Therefore, we specifically focused on these factors. For all selected aptamers, buffer optimization was performed by varying KCl concentration and pH, while the concentrations of NaCl and Tris-HCl were kept constant at 50 mM and 25 mM, respectively.

**Figure 2:**
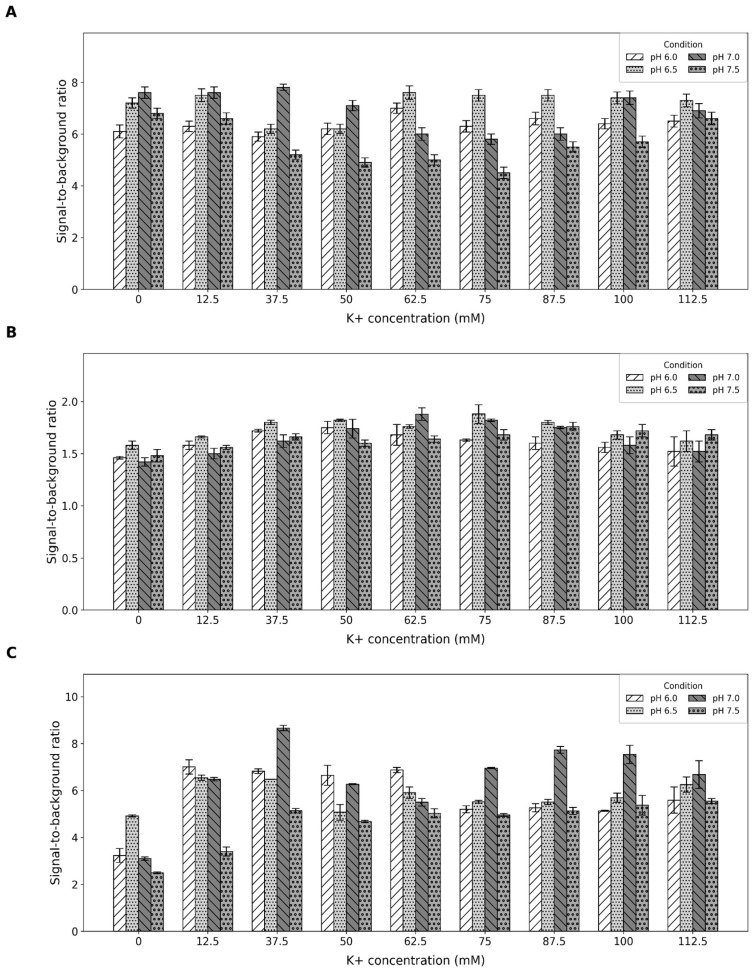
Optimization of reaction conditions for the CV30S aptamer (**A**), G4 parallel (**B**), and G4 antiparallel (**C**). The diagram represents how the S/B ratio depends on K^+^ concentration and solution pH. The x-axis indicates the K^+^ concentrations (0, 12.5, 37.5, 50, 62.5, 75, 87.5, 100, 112.5). Within each group, results are shown for four different pH values (6.0, 6.5, 7.0, 7.5). Error bars indicate standard deviation. All the experiments were conducted in triplicate.

For the CV30S aptamer and parallel G4, the optimal buffer was identified as one with pH 7 and 62.5 mM KCl, which yielded the highest S/B ratio of 7.8 (Figure 2A). The absolute fluorescence intensity of CV30S in this buffer was approximately 13,000 (Figure S1A). For the G4 parallel, the S/B ratio was 1.9 (Figure 2B), with the absolute fluorescence intensity around 3000 (Figure S1B) in the same buffer (pH 7; 62.5 mM KCl). The parallel G4 also exhibited a comparably high signal in a different buffer with a lower pH and higher KCl concentration (pH 6.5; 75 mM KCl), showing similar S/B ratio and absolute intensity. We selected the buffer (pH 7; 62.5 mM KCl).

For the G4 antiparallel aptamer, the optimal buffer composition was pH 7 and 37.5 mM KCl. Under these conditions, the S/B ratio was 8.7 (Figure 2C), and the absolute fluorescence intensity was around 14,500 (Figure S1C).

In all experiments for buffer optimization, we observed that the signal was lower in reactions with the parallel G4 compared to antiparallel G4, despite their structural similarity. This observation aligns with prior reports [35] and can be attributed to differences in G4 loop structures. According to the literature data, antiparallel G4 binds CV and retains molecules both on the side of the complex and within the aptamer structure [36].

### 2.2. Aptamer-to-Fluorescent Ratio

The efficiency of fluorophore binding by different aptamers can vary depending on reaction conditions. To identify the optimal aptamer-to-fluorophore ratio, we determined the maximal values of absolute fluorescence intensity and the S/B ratio at varying aptamer concentrations.

**Figure 3:**
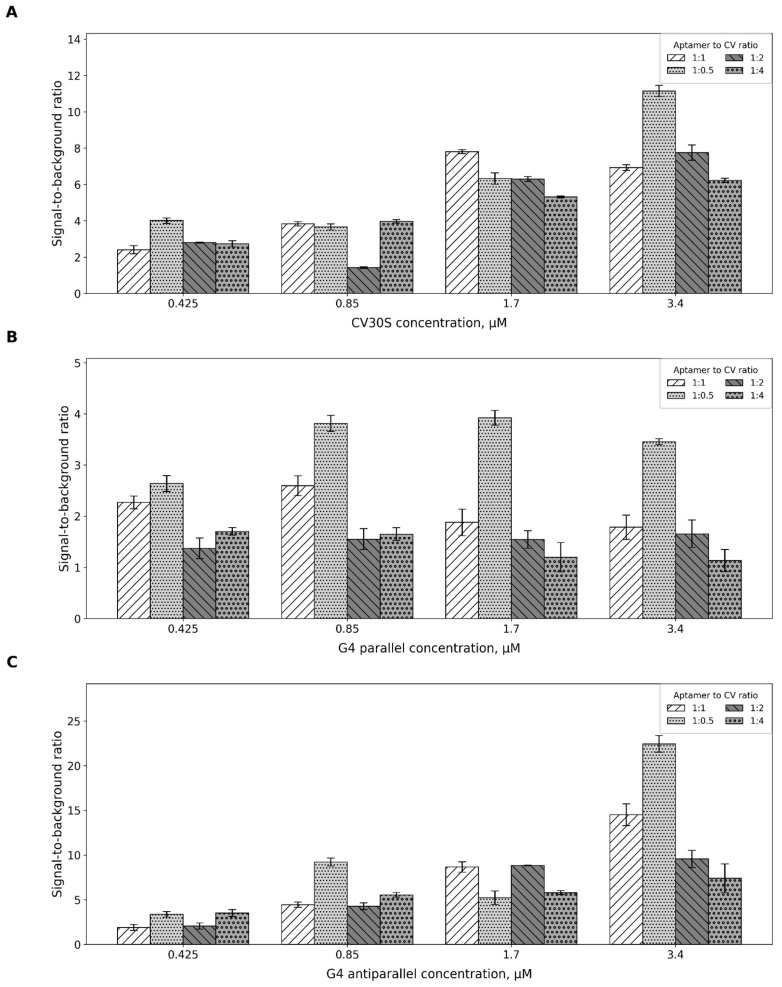
Optimization of the aptamer-to-CV ratio for the CV30S aptamer (**A**), G4 parallel (**B**), and G4 antiparallel (**C**). The diagram represents the dependence of the S/B on different concentrations of aptamers and aptamer-to-CV ratios. The x-axis groups data according to aptamer concentrations (0.425, 0.85, 1.7, 3.4 µM). Within each group, results are shown for four different aptamer-to-CV ratios (1:1, 1:0.5, 1:2, 1:4). Error bars represent standard deviation. All the experiments were conducted in triplicate.

Our findings indicate that for the CV30S aptamer, the optimal aptamer-to-CV ratio is 3.4 μM to 1.7 μM, yielding the highest S/B ratio of 10.2. Alternatively, a 1.7 μM to 1.7 μM ratio can also be used, providing the highest S/B ratio of ~7.8 (Figure 3A and Figure S2A). Both the G4 parallel and the G4 antiparallel aptamers require 1.7 μM to 0.85 μM aptamer-to-CV ratio to achieve the best signal (Figure 3B,C and Figure S2B,C). The main difference lies in the signal strength: S/B for the parallel G4 is around 3.9, while for the antiparallel G4 it reaches as high as 9. The same S/B ratio was achieved for the G4 antiparallel aptamer under different conditions: 1.7 μM: 1.7 μM or 1.7 μM: 3.4 μM. To reduce costs, we recommend using the 1.7 μM to 0.85 μM ratio.

For the final characterization, the fluorescence spectra of the aptamer–dye pairs were recorded under optimal conditions (Figure S3). These spectra confirm the appropriate selection of the excitation and emission wavelengths.

### 2.3. CV30S Aptamer Splitting

To develop aptamer-based binary biosensors, it is crucial to identify the nucleotide positions where the strand breaks can be introduced without significantly compromising their binding affinity to the dye molecule. Subsequently, two segments of the aptamer can be fused with different sequences, creating two strands of the sensor which can form a functional complex, consisting of two oligonucleotides and a dye (Figure 1B). These cleavage sites can often be predicted based on the known aptamer structure or derived from previous studies [37]. However, for the CV30S aptamer, this information was not available and had to be defined experimentally.

**Figure 4:**
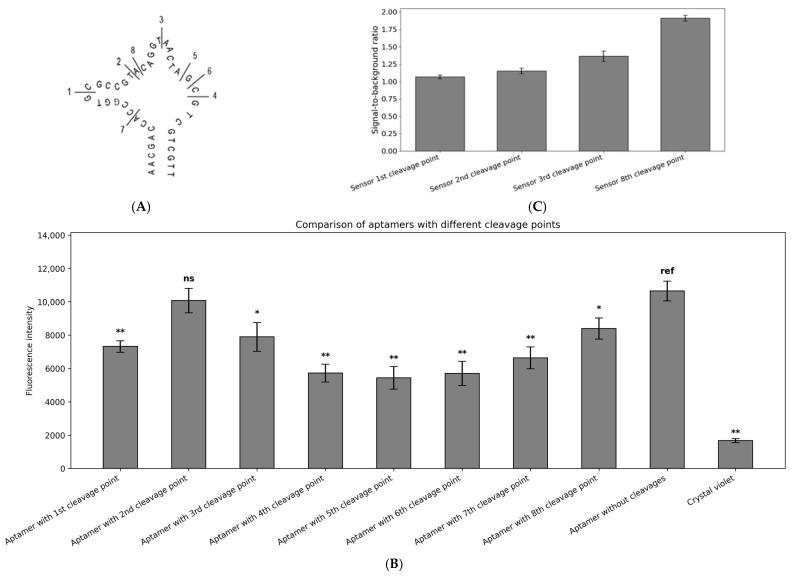
Identification of optimal CV30S cleavage sites. (**A**) Schematic representation of the cleavage sites introduced into the secondary structure of the CV30S aptamer. (**B**) Fluorescence intensities of aptamers split at different cleavage points (1st–8th, as indicated in Figure 4A), the intact aptamer without cleavages was used as a positive control. (**C**) S/B ratios obtained for selected binary sensors (cleavage points: 1st, 2nd, 3rd, and 8th). Error bars indicate standard deviation. Asterisks (*, **) indicate statistical significance, ns indicates no statistical significance, ref is the reference positive sample. All experiments were conducted in triplicate.

Eight positions within the CV30S aptamer were selected for introducing cleavage points (Figure 4A). All pairs of aptamer fragments were incubated with CV in the optimal buffer and at the previously identified optimal ratios. We have shown that cleavage of the CV30S at the site 2 yielded the best performance, followed by the cleavage at sites 8, 3, and 1 (Figure 4B). The pairs of aptamer fragments cleaved at sites 1, 2, 3, and 8 were subjected to further analysis as binary sensors.

Three CV30S-based binary sensors were designed, each featuring different cleavage points within the CV30S aptamer structure (Table S1 and Figure 4C and Figure S4). A fragment of the 16S gene from *Aeromonas hydrophila* was used as an analyte, with a reference temperature for the design process of 37 °C. Although the absolute fluorescence intensity was highest for the aptamer cleaved at site No. 2, its S/B ratio in a binary form was significantly low due to a high background of about 1, with an absolute fluorescence intensity around 16000. A similar issue was observed with the CV30S cleaved at site 1, which also exhibited an S/B ratio of ~1 and an absolute fluorescence of 10,000. Therefore, all further experiments were performed using the aptamer cleaved at site No. 8, which demonstrated an S/B ratio of 2 and an absolute fluorescence of 10,000.

### 2.4. Design of DNA Nanosensors

**Figure 5:**
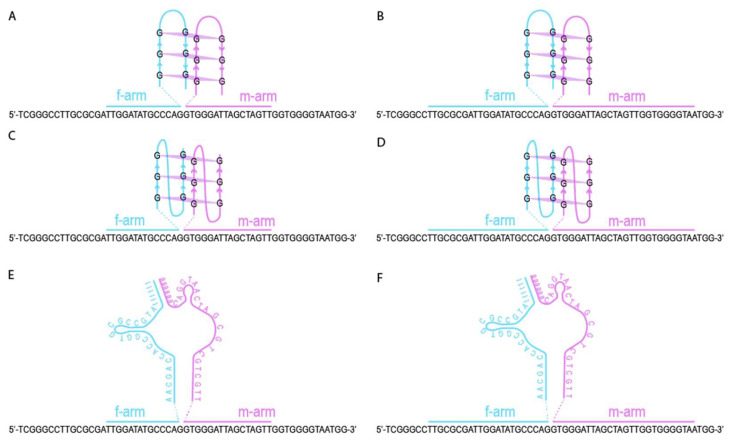
Sensors based on different aptamer core fused with short- and long-analyte-binding arms. (**A**) G4 parallel core with short-analyte-binding arms, (**B**) G4 parallel core with long-analyte-binding arms, (**C**) G4 antiparallel core with short-analyte-binding arms, (**D**) G4 antiparallel core with long-analyte-binding arms, (**E**) CV30S core with short-analyte-binding arms, and (**F**) CV30S core with long-analyte-binding arms.

Six binary sensors were designed on the base of G4 antiparallel, G4 parallel, and CV30S (split at the eighth cleavage site) cores. Each core was extended with analyte-binding arms tailored for reaction temperature 37 °C and 55 °C (Figure 5). The temperature values depend on the length of the analyte-binding arms and correlate with the temperature of the nucleic acid incubation. Before incubation, the assembled nucleic acid complexes are cooled down to room temperature, which is optimal for aptamer–dye signal generation.

The analyte-binding arms designed for 37 °C were relatively short and are referred to as ‘short’ (Figure 5A,C,E), while the analyte-binding arms designed for the 55 °C incubation were relatively long and are referred to as ‘long’ (Figure 5B,D,F). Details of the design process are described in the Section 4.

### 2.5. Optimization of Fragment Ratios in Binary Sensors

Nucleic acids in solution may adopt secondary structures or form dimers that decrease their availability to the sensing complexes. To achieve optimal performance, the ratio between fragments of the sensor (designated as f- and m-, see Figure 5) should be analyzed. The fragment combination with the highest S/B ratio proceeds to the limit of detection (LOD) test.

**Figure 6:**
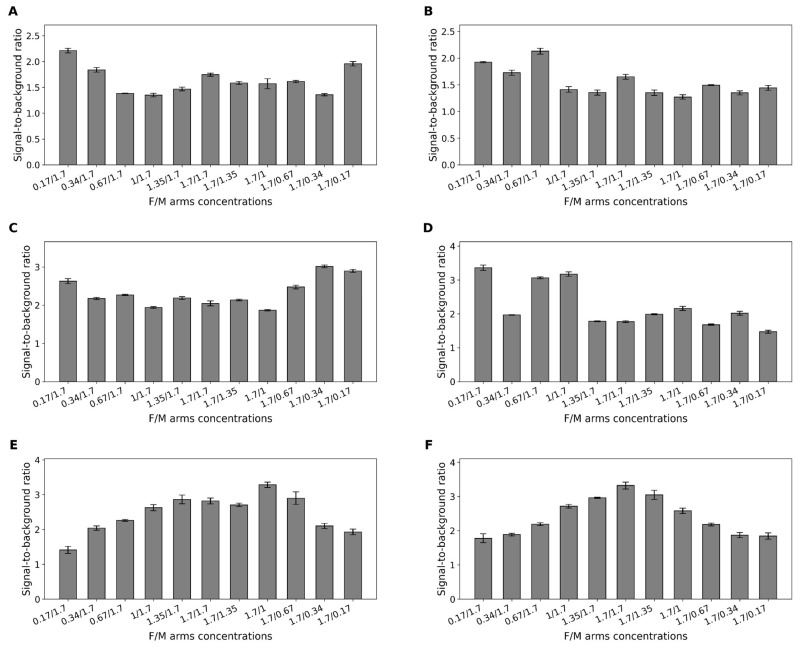
Optimization of m-/f-analyte-binding arms ratios for binary sensors. S/B ratios are shown for: (**A**,**B**) CV30S core with short and long arms; (**C**,**D**) G4 parallel core with short and long arms; and (**E**,**F**) G4 antiparallel core with short and long arms. Error bars indicate standard deviation. All the experiments were conducted in triplicate.

The results indicated that for the sensor based on the CV30S aptamer, the optimal ratio of arms is 0.17:1.7 µM for the short-armed sensor and 0.67:1.7 µM for the long-armed sensor, achieving the highest S/B ratios of 2.2 and 2.1, respectively (Figure 6A,B and Figure S5A,B). The best performance of the G4 parallel was observed at a ratio of 1.7: 0.34 µM for the short-armed sensor and 0.17: 1.7 µM for the long-armed sensor, yielding S/B ratios of 3 and 3.3, respectively (Figure 6C,D and Figure S5C,D). The best performance of the G4 antiparallel aptamer was observed at a ratio of 1.7: 1.35 µM for the short-armed sensor and 1.7: 1.7 µM for the long-armed sensor, yielding S/B ratios of 3.2 and 3.3, respectively (Figure 6E,F and Figure S5E,F).

The findings show that incorporating G4 into the binary sensor mitigates the high values previously observed with the whole antiparallel G4 configuration discussed in Section 2.2. When split, both the parallel and antiparallel configurations tend to generate S/B ratios as low as approximately 3.

### 2.6. Limit of Detection (LOD)

**Figure 7:**
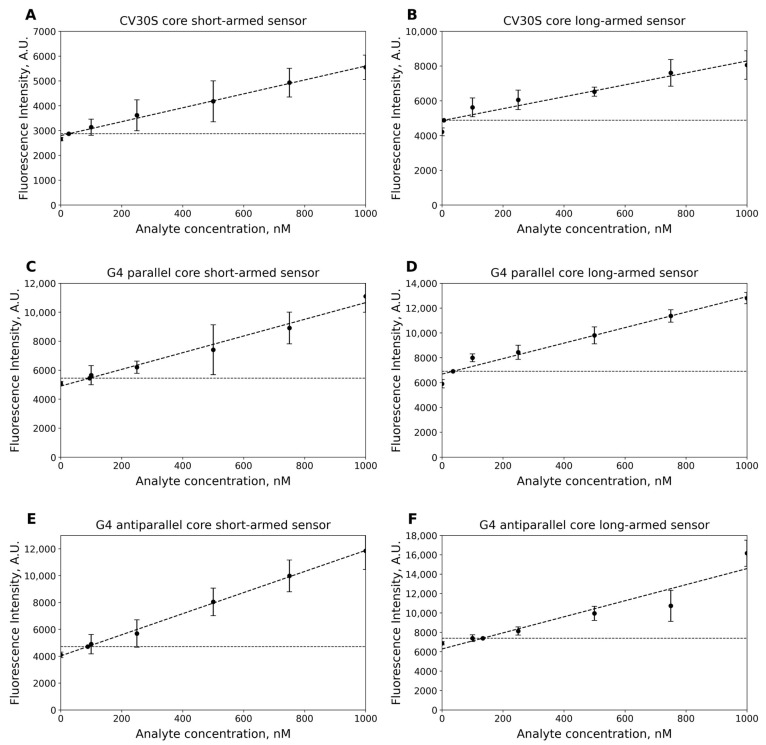
Limits of detection of the CV sensors after 30 min of incubation: (**A**) CV30S core short-armed sensor; (**B**) CV30S core long-armed sensor; (**C**) G4 parallel core short-armed sensor; (**D**) G4 parallel core long-armed sensor; (**E**) G4 antiparallel core short-armed sensor; and (**F**) G4 antiparallel core long-armed sensor. Error bars indicate standard deviation. All the experiments were performed in triplicates. The inclined lines present the linear regressions, while the horizontal lines are the threshold of the three standard deviations of the blank above the signal. The LOD is at the intersection of the linear regression and the threshold.

The limit of detection (LOD) refers to the lowest concentration that can be reliably identified, indicating it is greater than zero. This metric is commonly used in biosensor development to determine the threshold at which a signal can be statistically distinguished from a blank sample [38]. The LOD for the G4 parallel-based binary sensors was found to be 94.5 nM and 36.4 nM for the short-armed and long-armed sensors, respectively, after 30 min of incubation (Figure 7A,B). For the G4 antiparallel-based binary sensors, the LOD was found to be 91.9 nM and 125.9 nM for the short-armed and long-armed sensors, respectively, also after 30 min of incubation (Figure 7C,D). The LOD for the CV30S-based binary sensors was determined at 68.5 nM and 6 nM for the short-armed and long-armed sensors, respectively, again after 30 min of incubation (Figure 7E,F).

While the CV30S-based sensors have the lowest S/B ratio, they demonstrated drastically reduced LOD in the long-armed configuration. Conversely, the G4 antiparallel short-armed configuration performed well at 37 °C. This may be caused by higher dissociation coefficient of the CV–aptamer complex, that could be beneficial at these temperatures.

## 3. Discussion

In this study, we systematically compared three previously reported CV-binding aptamers: CV30S, G4 parallel, and G4 antiparallel. We evaluated their ability to generate fluorescence, their specific S/B ratio, and their potential as cores for binary sensors. Our results demonstrate that each of the CV30S and G4 antiparallel aptamers possess distinct advantages that may be useful for different applications in various analyte detection. For example, the LOD of the CV30S aptamer is suitable for amplification-free detection of conditionally pathogenic *Aeromonas hydrophila*, which is characterized by distinct levels of insemination.

Among the studied aptamers, the G4 antiparallel structure consistently demonstrated the highest absolute fluorescence intensity and robustly high S/B ratio in solution, confirming previous reports that G-quadruplex topologies can efficiently stabilize the CV binding and enhance fluorescence. In contrast, the G4 parallel aptamer showed the lowest performance, both in terms of intensity and sensitivity, suggesting that its structural topology is less favorable for efficient G4–CV interaction under the tested conditions.

Unexpectedly, the non-G4 CV30S aptamer displayed unique properties when incorporated into binary sensors. While its fluorescence in solution was comparable or slightly inferior to G4 aptamers, the binary sensor configurations based on CV30S achieved the best LOD (as low as 6 nM). This finding indicates that CV30S may undergo favorable structural rearrangements upon inclusion into binary sensors, enhancing the signal readout compared to its free aptamer form.

The superior detection limits of CV30S-based binary sensors highlight their potential for sensitive biosensing applications. The low detection limit is likely associated with high dissociation coefficient, which means that once the complex is formed, it does not disassemble easily. However, the relatively low S/B ratio in this case reflects challenges with the CV–CV30S complex formation. Importantly, our results on the cleavage site optimization revealed that non-intuitive design choices (e.g., sites 2 and 8 being separated by only one nucleotide yet exhibiting drastically different performance) could further improve sensor efficacy. This underscores the necessity of empirical testing during aptamer-based sensor engineering [39].

Both G4 aptamers also demonstrated better S/B ratio; therefore, these aptamers, with higher signal magnitudes, may be more suitable for use in selectivity testing. They could be particularly relevant for single nucleotide polymorphism (SNP) detection, especially in cases where significant differences in low signal values may not exist [40]. However, future investigations will be required to validate this application.

Overall, our study demonstrates that while G4 antiparallel aptamers provide strong signal intensity and reliability, the CV30S aptamer shows superior detection sensitivity when included into binary sensors. This duality suggests that aptamer choice should be guided by the specific application requirements—high intensity versus low detection limit. Future work may focus on structural analysis of CV30S to elucidate the molecular basis of its enhanced nanosensor performance and to extend its use toward practical diagnostic platforms due to its higher selectivity for the dye.

## 4. Materials and Methods

### 4.1. Materials

The Aptamer buffer contains MgCl_2_ (AppliChem, Darmstadt, Germany), Tris (Amresco, Solon, OH, USA), KCl (Carl Roth, Karlsruhe, Germany), and NaCl (Vekton, Saint Petersburg, Russia). Crystal violet was purchased from Lenreactiv, Russia. RNase/DNase-free sterile water for dilutions was purchased from Evrogen, Russia. All the oligonucleotides were purchased from Evrogen, Russia, via direct order.

The fluorescence measurement was made via Spark reader (Tecan, Mannedorf, Switzerland). Nucleic acid concentration measurements were made via N50 (Implen, Munich, Germany).

### 4.2. Buffer Optimization

To 1.9 µL of CV solution (100 µM), 1.9 µL of aptamer solution (100 µM) was added, along with one of several buffer variants differing in KCl concentration and pH, resulting in a final reaction volume of 110 µL. The buffers are stable in terms of Tris-HCl concentration—25mM—and NaCl concentration—50mM. The pH was optimized using HCl at values from 6.0 to 7.5, while KCl concentration was varied in the range of 0 to 112.5 mM.

The samples were incubated for 15 min at 55 °C to disrupt potential interfering secondary structures in the analyte, followed by a 10 min incubation at room temperature (~22 °C) to cool down and set the binding. After incubation, the samples were transferred to a black 96-well plate. The final concentrations of aptamer and CV in the mixture were both 1.7 µM, with a total reaction volume of 110 µL. Fluorescence was measured using a Spark plate reader (Tecan, Mannedorf, Switzerland) at excitation/emission wavelengths of 580 nm/640 nm.

### 4.3. Aptamer-to-CV Ratio

To 0.55 µL, 1.1 µL, 2.2 µL, and 4.4 µL of aptamer solution (100 µM), CV was added to achieve the following ratios: 1:0.5, 1:1, 1:1.5, 1:2, and 1:2.5. The reactions were brought to a final volume of 130 µL. The final concentrations of CV in the mixture were 0.42 µM, 0.85 µM, 1.7 µM, and 3.38 µM, respectively.

### 4.4. Aptamer Splitting

The oligonucleotides CV30S_mX and CV30S_fX (where X is the cleavage site number) were added to the samples according to the optimized buffer conditions (Section 2.1) and the aptamer-to-fluorescent ratio (Section 2.2). Samples were incubated at 37 °C or 55 °C for 30 min. A positive control consisted of the intact CV30S aptamer, while the negative control contained only the dye in the buffer. After incubation, the samples were transferred to a black 96-well plate for fluorescent measurement. This measurement was performed using a Spark fluorimeter (Tecan, Switzerland) at excitation/emission wavelengths of 580 nm/640 nm.

### 4.5. Binary Sensor Design

A panel of binary sensors was developed using aptamers as a split core. A fragment of the 16S rRNA gene of *Aeromonas hydrophila* (CP046954.1) was used as an analyte. The design of the binary sensors was carried out using the web applications UNAFold (https://www.unafold.org/, accessed on 25 April 2025) and the OligoAnalyzer Tool (https://www.idtdna.com/calc/analyzer, accessed on 25 April 2025) [40]. The following guidelines were considered to ensure the successful design of the sensor arms [41]:

1. While selecting the recognition site for the biosensor, the secondary structure of the target DNA region must be taken into account.
2. If the target DNA sequence folds into a complex secondary structure forming a loop, the sensor arms should be placed on the same side of the loop.
3. The melting temperature should be within the range of 35–40 °C for detection at 37 °C or 50–60 °C for detection at 55 °C.
4. Each sequence should be linked to one of the aptamer sequences through a T-linker composed of two to six thymidine residues.
5. Each sensor arm must be checked for secondary structure formation at 37 °C and 55 °C. They should not form loop structures, and the overall Gibbs free energy of the structure should not exceed -4 kcal/mol.

### 4.6. Binary Sensor Arm Ratio Optimization

The corresponding oligonucleotides, aptamer_f and aptamer_m (where ‘aptamer’ refers to the type of aptamer in the structure of binary sensors), were added to the sample at different concentrations in µM (1.7/1.7 (equal, Ctrl); 1.35/1.7; 1.0/1.7; 0.67/1.7; 0.34/1.7; 0.17/1.7; 1.7/0.17; 1.7/0.34; 1.7/0.67; 1.7/1.0; 1.7/1.35) according to the optimized buffer conditions (Section 2.1) and the aptamer-to-CV conditions (Section 2.2). Analytes were added at a concentration of 1 µM. Samples were incubated following the protocol outlined in Section 4.4, at 37 °C or 55 °C for 30 min. The positive control consisted of the intact aptamer, while the negative control was the dye in the buffer. After incubation, samples were transferred to a black 96-well plate for fluorescent measurement, which was performed using a Spark fluorimeter (Tecan, Switzerland) at excitation/emission wavelengths of 580 nm/640 nm.

### 4.7. Limits of Detection

Each sample was prepared in 65 µL of 2X optimal buffer (see Section 2.1) containing the optimal concentration of CV and optimal arm concentrations (see Section 2.2), along with a specific synthetic analyte in concentrations ranging from 0 nM to 1 µM. The final reaction volume was brought to 110 µL. Samples were incubated at 37 °C or 55 °C for 30 min (for short and long sensors, respectively). A positive control consisted of the intact aptamer, while the negative control was the CV dye in the buffer. After incubation, samples were again transferred to a black 96-well plate for fluorescent measurement, which was performed using a Spark fluorimeter (Tecan, Switzerland) at excitation/emission wavelengths of 580 nm/640 nm. LOD was quantified as the cross section of a linear regression and three SD above the mean of the blank [38].

## 5. Conclusions

A systematic analysis of the CV’s aptamer performance was carried out. The binary sensors based on CV30S presented low LOD, while the antiparallel G4 core suggested a high S/B ratio. The LOD is likely due to a high dissociation coefficient preventing the complexes from disassembling. Relatively high S/B ratio of the antiparallel G4–CV aptamer–dye systems suggests the favorability of complex formation in comparison to the CV30S and antiparallel G4 designs. The parallel G4 core was the least successful choice for the biosensor development.

## Data Availability

The raw data are available upon request.

## Supplementary Materials

The following supporting information can be downloaded at: https://www.mdpi.com/article/10.3390/ijms26199833/s1.

## Abbreviations

The following abbreviations are used in this manuscript:

CV	Crystal Violet
G4	G-Quadruplex
LOD	Limit of Detection
S/B	Signal-to-Background
