# Supplementary material for: Comparative Analysis of Crystal Violet-Binding Aptamers as Potential Cores for Binary Sensors
Source: Int J Mol Sci. 2025 Oct 9;26(19):9833. doi: 10.3390/ijms26199833 (PMC12524621; doi:10.3390/ijms26199833)
Supplement: Supplementary file 1 [file ijms-26-09833-s001.zip › ijms-3904127-supplementary.pdf]

Supplementary Materials

Comparative Analysis of Crystal Violet-Binding Aptamers as Potential Cores for Binary Sensors

Gleb A. Bobkov [1,2,3,†], Gleb S. Yushkov [2,†], Andrei D. Kuzmin [1,2], Tatiana D. Popysheva [2], Elena I. Stepchenkova [1,3,*] and Maria S. Rubel [1,2,*]

[1] Saint-Petersburg State University, 199034 Saint Petersburg, Russia; gleb.bobkov@spbu.ru (G.A.B.); andrei.kuzmin.itmo@gmail.com (A.D.K.)
[2] ITMO University, 191002 Saint Petersburg, Russia; yushkov@scamt-itmo.ru (G.S.Y.); tanya.popyscheva@yandex.ru (T.D.P.)
[3] Vavilov Institute of General Genetics, Saint Petersburg Branch, Russian Academy of Sciences, 199034 Saint Petersburg, Russia
* Correspondence: e.stepchenkova@spbu.ru (E.I.S.); m.rubel@spbu.ru (M.S.R.)
† These authors contributed equally to this work.

Table S1. The Oligonucleotides used in research.

Oligonucleotide Name	Sequence
Analyte	TCGGGCCTTGCGCGATTGGATATGCCCAGGTGGGATTAGCTAGTTGGTGGGGTAATGG
CV30S_8_m_short	AACTAGCTAATCCCACTTTAACGACCACCGGTGCGCCGTATTTTTT
CV30S_8_f_short	AAAAAACAGGTAACTAGCGTCGTCGTTTTTCTGGGCATATCCAA
CV30S_8_m_long	TTACCCCACCAACTAGCTAAT CCC ACTTTAACGACCACCGGTGCGCCGTATTTTTT
CV30S_8_f_long	AAAAAACAGGTAACTAGCGTCGTCGTTTTTCTGGGCATATCCAATCGCGCAA
G4antiparallel_m_short	AACTAGCTAATCCCACTTTTTTAGGGTTAGG
G4antiparallel_f_short	GTTAGGGTTAGGGTTTTTTCTGGGCATATCCAA
G4antiparallel_m_long	TTACCCCACCAACTAGCTAAT CCC ACTTTTTTAGGGTTAGG
G4antiparallel_f_long	GTTAGGGTTAGGGTTTTTTCTGGGCATATCCAATCGCGCAA
G4parallel_m_short	AACTAGCTAATCCCACTTTTTTGGGTAGGG
G4parallel_f_short	GGGTTGGGTTTTTTCTGGGCATATCCAA
G4parallel_m_long	TTACCCCACCAACTAGCTAAT CCC ACTTTTTTGGG TAG GG
G4parallel_f_long	GGG TTG GG TTTTTT CTGGGCATATCCAATCGCGCAA
Aptamer_G4_antiparallel	AGGGTTAGGGTTAGGGTTAGGG
Aptamer_G4_parallel	GGG TAG GGC GGG TTG GG
Aptamer_CV30S	AACGACCACCGGTGCGCCGTACAGGTAACTAGCGTCGTCGTT
CleavedCV30S_1_m	AACGACCACCGGTGTTTTTT

CleavedCV30S_1_f	AAAAAACGCCGTACAGGTAACTAGCGTCGTCGTT
CleavedCV30S_2_m	AACGACCACCGGTGCGCCGTTTTTTT
CleavedCV30S_2_f	AAAAAAACAGGTAACTAGCGTCGTCGTT
CleavedCV30S_3_m	AACGACCACCGGTGCGCCGTACAGGTTTTTTT
CleavedCV30S_3_F	AAAAAAAACTAGCGTCGTCGTT
CleavedCV30S_4_m	AACGACCACCGGTGCGCCGTACAGGTAACTAGCTTTTTT
CleavedCV30S_4_f	AAAAAAGTCGTCGTT
CleavedCV30S_5_m	AACGACCACCGGTGCGCCGTACAGGTAACTATTTTTT
CleavedCV30S_5_f	AAAAAAGCGTCGTCGTT
CleavedCV30S_6_m	AACGACCACCGGTGCGCCGTACAGGTAACTAGTTTTTT
CleavedCV30S_6_f	AAAAAACGTCGTCGTT
CleavedCV30S_7_m	AACGACCACCGGTGCGCCGTACAGGTAACTAGCTTTTTT
CleavedCV30S_7_f	AAAAAAGTCGTCGTT
CleavedCV30S_8_m	AACGACCACCGGTGCGCCGTATTTTTT
CleavedCV30S_8_f	AAAAAACAGGTAACTAGCGTCGTCGTT

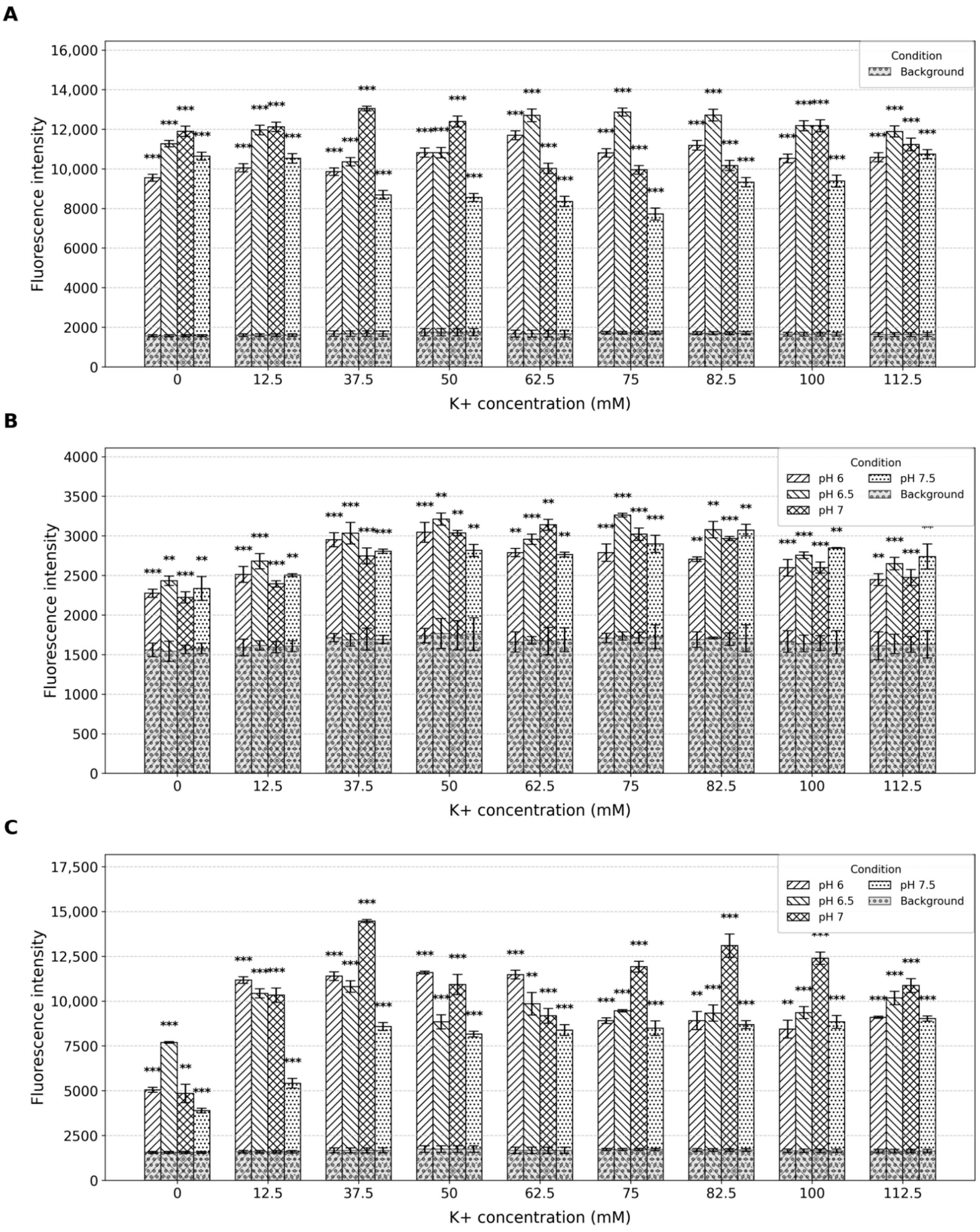

Figure S1. Optimization of reaction conditions for the CV30S aptamer (**A**), G4 parallel (**B**), and G4 antiparallel (**C**), shown as absolute fluorescence and background values as a function of K^+ concentration and solution pH. The x-axis is grouped by K^+ concentration (0, 12.5, 37.5, 50, 62.5, 75, 82.5, 100, 112.5); within each group, results are shown for four pH values (6.0, 6.5, 7.0, 7.5). Error bars indicate standard deviation (n = 3). Asterisks indicate statistical significance.

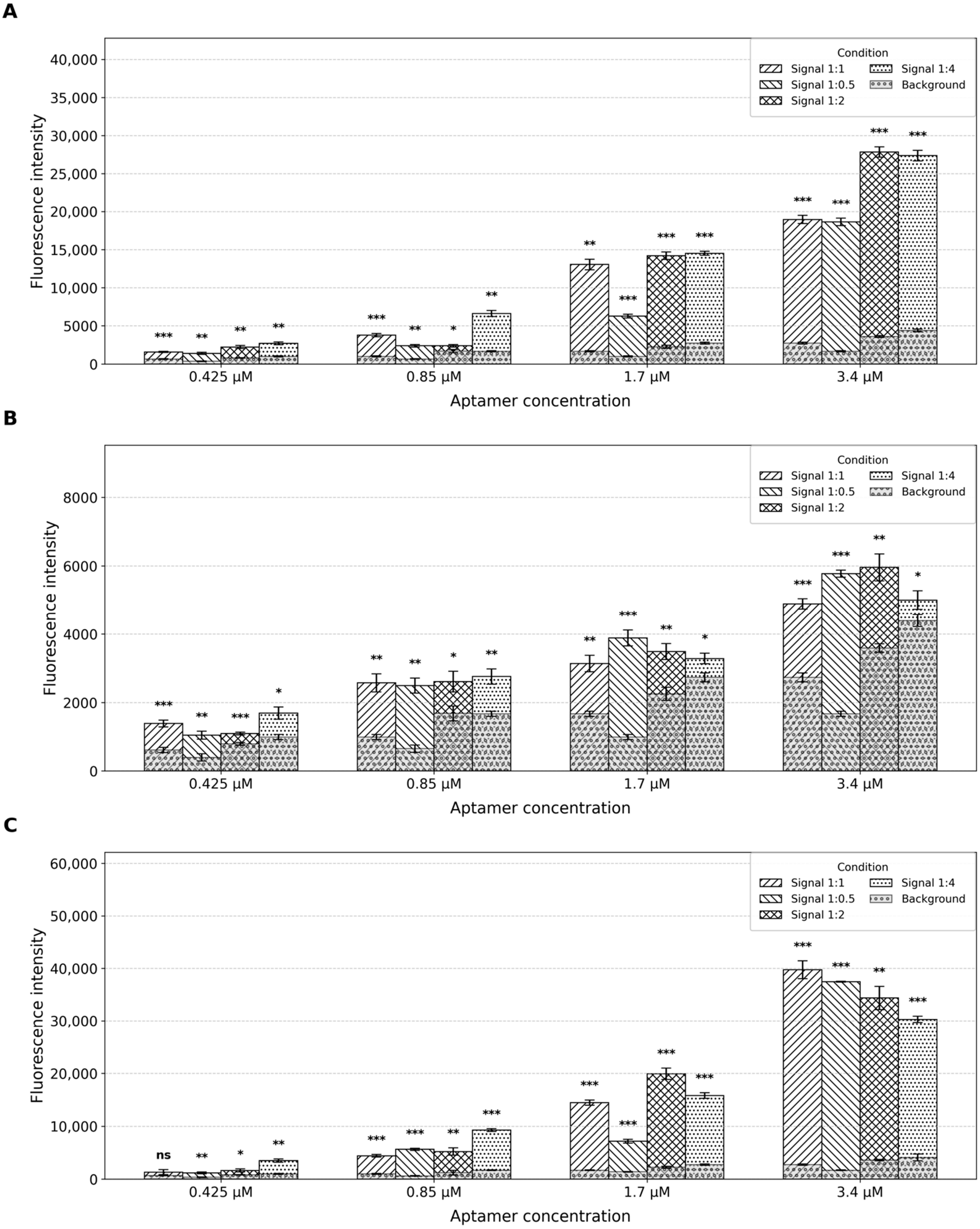

Figure S2. Optimization of aptamer and CV concentrations for the CV30S aptamer (**A**), G4 parallel (**B**), and G4 antiparallel (**C**), shown as absolute signal and background fluorescence as a function of aptamer concentration and aptamer-to-CV ratio. The x-axis is grouped by aptamer concentration

(0.425, 0.85, 1.7, 3.4 μM); within each group, results are shown for four aptamer-to-CV ratios (1:1, 1:0.5, 1:2, 1:4). Error bars indicate standard deviation (n = 3). Asterisks indicate statistical significance.

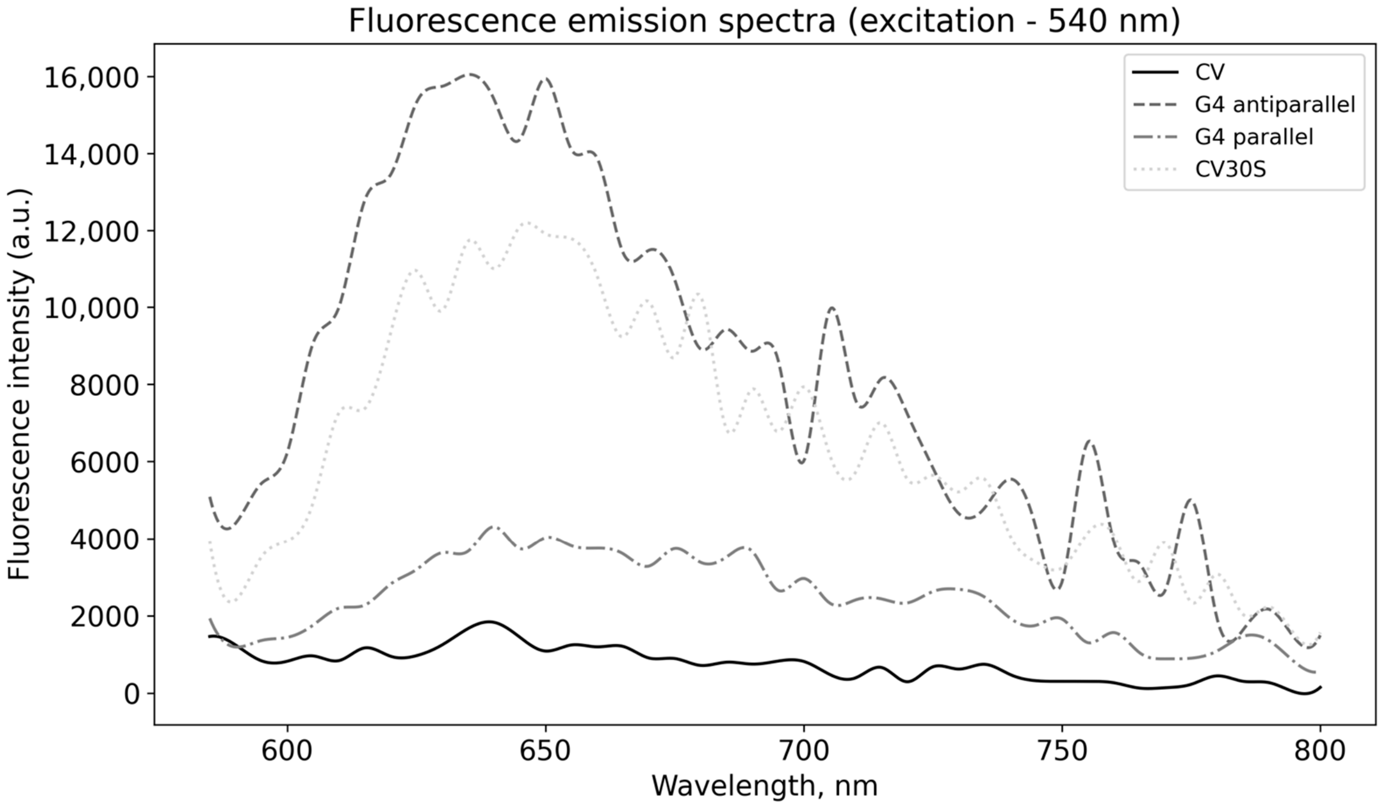

Figure S3. Fluorescence spectra of CV emission with the aptamers at optimum concentration and ionic conditions. The excitation wavelengths is 540 nm.

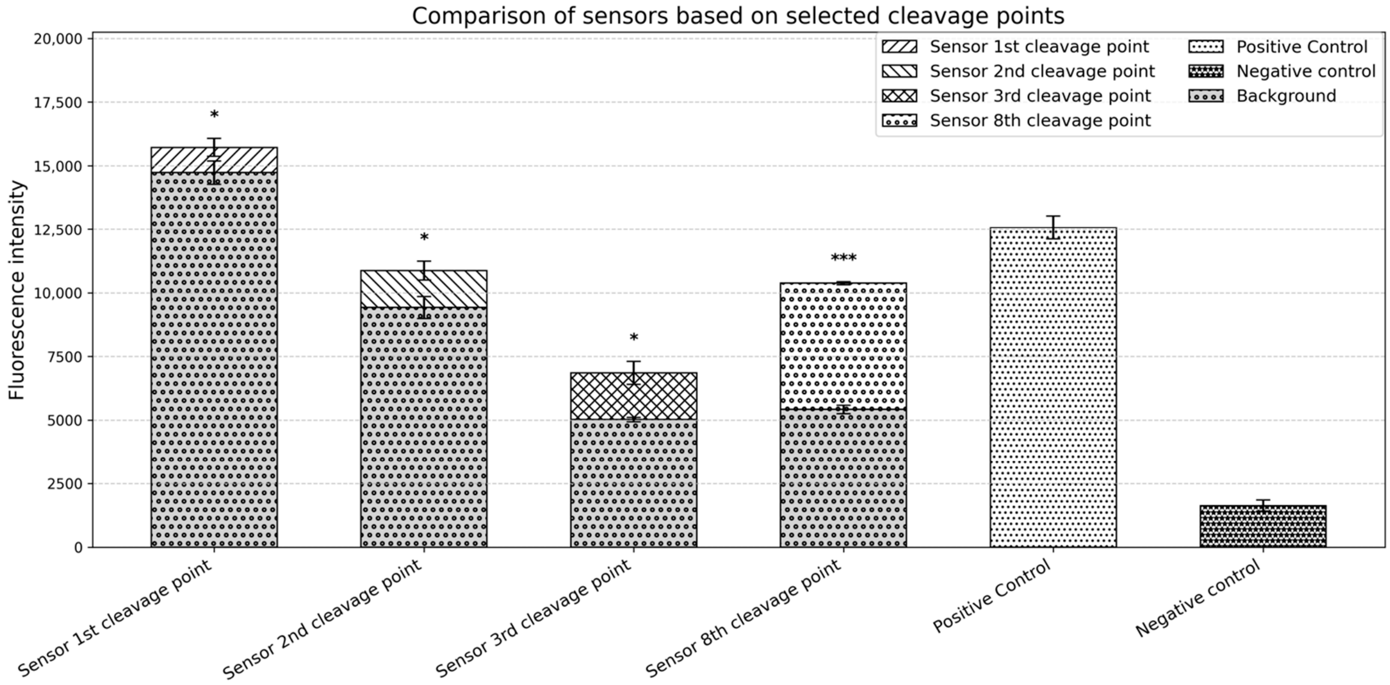

Figure S4. Absolute and background fluorescence of binary sensors constructed from the best-performing cleavage points (1st, 2nd, 3rd, and 8th). Error bars indicate standard deviation. Asterisks indicate statistical significance. All the experiments were performed in triplicates.

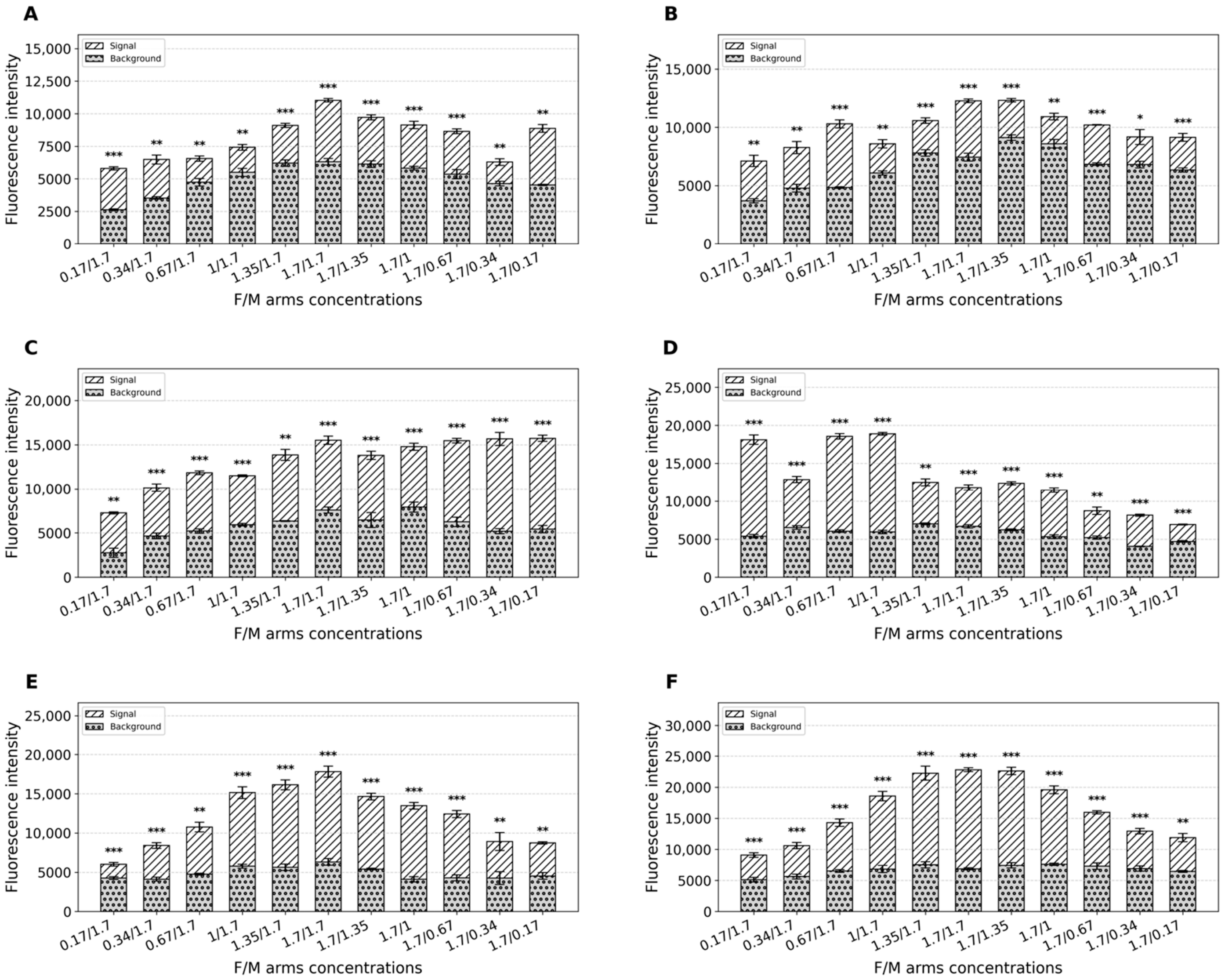

Figure S5. Optimization of m-/f-analyte-binding arm ratios for binary sensors, shown as absolute fluorescence and background values. (**A**,**B**) CV30S core with short and long arms; (**C**,**D**) G4 parallel core with short and long arms; (**E**,**F**) G4 antiparallel core with short and long arms. Error bars represent standard deviations. Asterisks indicate statistical significance. All experiments were performed in triplicates.
